# Impact of lignans in oilseed mix on gut microbiome composition and enterolignan production in younger healthy and premenopausal women: an in vitro pilot study

**DOI:** 10.1186/s12934-020-01341-0

**Published:** 2020-04-03

**Authors:** Giulia Corona, Anna Kreimes, Monica Barone, Silvia Turroni, Patrizia Brigidi, Enver Keleszade, Adele Costabile

**Affiliations:** 1 Health Science Research Centre, Department of Life Sciences, University of Roehampton, London, UK; 2 Unit of Holobiont Microbiome and Microbiome Engineering, Department of Pharmacy and Biotechnology, University of Bologna, Bologna, Italy

**Keywords:** Flaxseed, Enterolignans, Enterolactone, Enterodiol, Gut microbiome, Metabolism

## Abstract

**Background:**

Dietary lignans belong to the group of phytoestrogens together with coumestans, stilbenes and isoflavones, and themselves do not exhibit oestrogen-like properties. Nonetheless, the gut microbiota converts them into enterolignans, which show chemical similarity to the human oestrogen molecule. One of the richest dietary sources of lignans are oilseeds, including flaxseed. The aim of this pilot study was to determine the concentration of the main dietary lignans in an oilseed mix, and explore the gut microbiota-dependent production of enterolignans for oestrogen substitution in young and premenopausal women. The oilseed mix was fermented in a pH-controlled batch culture system inoculated with women’s faecal samples. The lignan content and enterolignan production were measured by ultra‐high-performance liquid chromatography–tandem mass spectrometry (UHPLC–MS/MS), and the faecal-derived microbial communities were profiled by 16S rRNA gene-based next-generation sequencing.

**Results:**

In vitro batch culture fermentation of faecal samples inoculated with oilseed mix for 24 h resulted in a substantial increase in enterolactone production in younger women and an increase in enterodiol in the premenopausal group. As for the gut microbiota, different baseline profiles were observed as well as different temporal dynamics, mainly related to *Clostridiaceae*, and *Klebsiella* and *Collinsella* spp.

**Conclusions:**

Despite the small sample size, our pilot study revealed that lignan-rich oilseeds could strongly influence the faecal microbiota of both younger and premenopausal females, leading to a different enterolignan profile being produced. Further studies in larger cohorts are needed to evaluate the long-term effects of lignan-rich diets on the gut microbiota and find out how enterolactone-producing bacterial species could be increased. Diets rich in lignans could potentially serve as a safe supplement of oestrogen analogues to meet the cellular needs of endogenous oestrogen and deliver numerous health benefits, provided that the premenopausal woman microbiota is capable of converting dietary precursors into enterolignans.
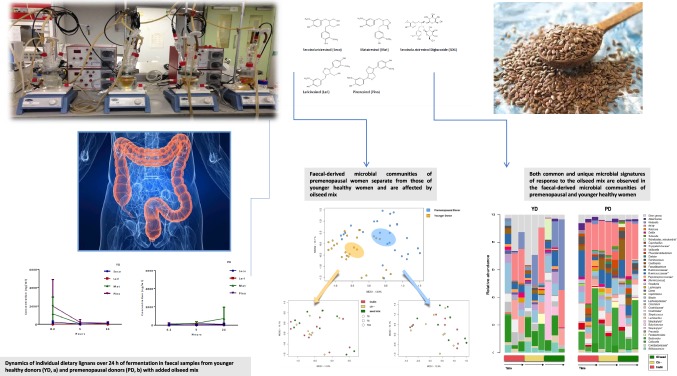

## Background

**Fig. 1:**
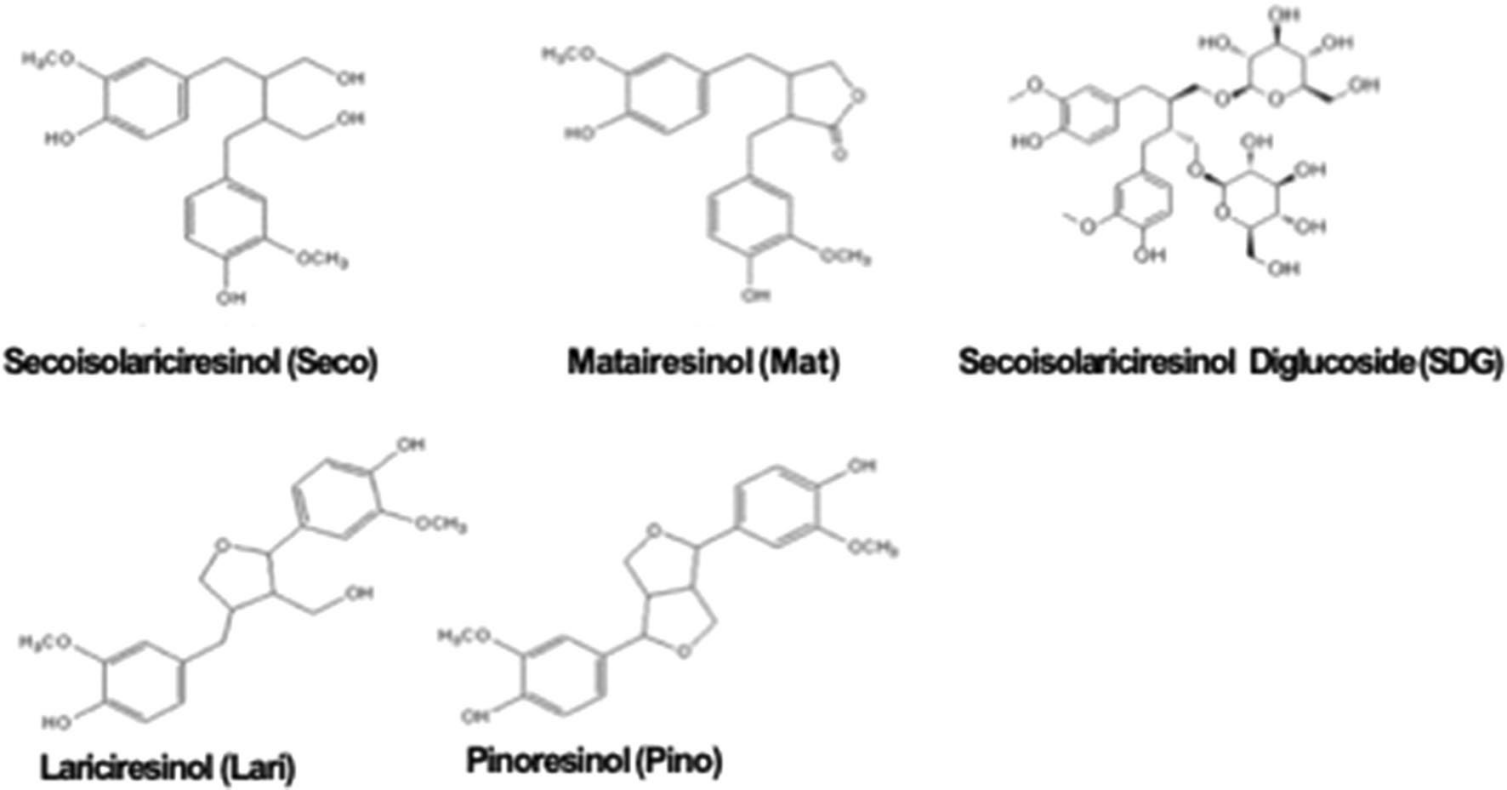
Dietary lignans. Secoisolariciresinol (Seco); Matairesinol (Mat); Secoisolariciresinol Diglucoside (SDG); Lariciresinol (Lari); Pinoresinol (Pino)

Dietary lignans are phytoestrogens found in plants as aglycones or glycosides [1]. Around 70 different plant species are rich in various lignans in their roots, rhizomes, stems, leaves, seeds, and fruits [2]. In particular, grains with bran and oilseeds, especially flaxseeds, are extremely rich sources [3, 4]. The most studied aglycone forms of dietary lignans are secoisolariciresinol (Seco), lariciresinol (Lari), pinoresinol (Pino) and matairesinol (Mat) (Fig. 1), whose range of concentration varies from 88.06 mg/100 g of fresh weight (FW) to 436.99 mg/100 g FW [3]. Secoisolariciresinol diglucoside (SDG), i.e. the glycosylated form of Seco, represents approximately 1% of the dry weight of flaxseeds [3], and its concentration ranges from 11.9 to 25.9 mg/g FW [5]. This has led to flaxseed becoming the most common source of enterolignan precursors for research.

Dietary lignans do not exhibit oestrogen-like properties but are known to be metabolised by the gut microbiota into enterolignans (or mammalian lignans), i.e. enterodiol (ED) and enterolactone (EL). Given that EL, more than ED, is able to bind the oestrogen receptor ER-α [6–9] and modulate endogenous oestrogen levels [10], it would be interesting to investigate the production of enterolignans by premenopausal-related gut microbiota layouts, following a lignan-rich diet. The magnitude of the modulating effects of enterolignans is expected to be determined by the biological level of endogenous oestrogen: enterolignans would exert antagonistic activity with normal oestrogen levels, while acting as weak oestrogens when their levels are low [11]. Additionally, it has been suggested that circulating enterolignans may stimulate the synthesis of sex hormone-binding globulin, which binds sex hormones, reducing their systemic levels and biological activity and, consequently, the risk of hormone-dependent cancer [12]. EL may also inhibit aromatase, an enzyme that converts androgens to oestrogens in fat, muscle and other tissues [13]. There is not much known yet about ER-β and G protein-coupled estrogen receptor (GPR), except that the deficiency of ER-β can inhibit expression of facilitative glucose transporter type 4 GLUT4 [14] and GPR is involved in body weight regulation, glucose and lipid homeostasis and inflammation (Sharma et al. [15]). After activating these receptors, oestrogen can promote mitotic activity, neoplastic transformation and cellular proliferation. The abundance of intracellular ER receptors confirms the significance of oestrogen and explains the fluctuations of the main physiological parameters during the natural female cycle as well as the dramatic health changes that follow the general decline in oestrogen after the menopause, such as an increase of cardiovascular conditions, weight gain, breast- and colon cancers, osteoarthritis and memory loss in post-menopausal women [16–18]. Suggesting that enterolignans are able to ligate on ER-α, substituting endogenous oestrogen and potentially alleviating the symptoms of lowered oestrogen, it would be interesting to investigate the potential role of a diet high in enterolignan precursors together with the activity of the gut microbiota in premenopausal women.

Numerous clinical trials and animal model studies have demonstrated the association between high concentrations of ED and EL in blood and urine and various health benefits, such as normal blood lipids [19], reduced risk of breast cancer [20] and osteoporosis [9, 21], as well as improved signs of metabolic syndrome [22]. In particular, a number of studies have revealed that the anti-cancer effect of enterolignans is higher than that of their precursors (i.e. dietary lignans) and that the decreased risk of cancer correlates with blood and urinary levels of enterolignans but not with lignan-rich diets [23–25], thus emphasizing the relevance of the gut microbiota as a key mediator of their effect.

According to in vitro studies from Peterson et al. [26], the faecal microbiota can metabolise 100% of Lari, producing 46% of EL and 54% of ED, while the microbial conversion of other lignans has been reported as incomplete (in 24 h 72% of SDG, 55% of Pino and 62% of Mat are metabolised into enterolignans, and only 21% of SDG will be converted into EL). Furthermore, some intestinal microbes have been shown to convert most ED to EL [27], but one-third of adults’ microbiotas could directly produce EL, skipping the ED stage [28]. As for gender, the female microbiota is capable of producing more ED and EL than the male one [29]. Even age has been reported to affect enterolignan production, with the child microbiota being less capable of converting dietary lignans into ED and EL [30].

Although the lignan transformation by the gut microbiota is recognized as essential in protecting against menopausal symptoms as well as certain hormone-dependent chronic diseases (e.g. cancer, cardiovascular disease and osteoporosis) [31–33], to date only a few bacterial species (often subdominant) have been identified as enabling such a transformation [33, 34]. In particular, EL production has been related to the abundance of *Ruminococcus* species, i.e. *R. bromii* and *R. lactaris* [35], as well as to those of *Lactobacillus*–*Enterococcus* [36] and *Methanobrevibacter*, an archaeon central to the syntrophic hydrogen metabolism that may be important for EL production [37]. On the other hand, low-EL excreter phenotypes have been associated with the pathobiont, sulphite-reducing bacterium, *Bilophila*, as well as microbial signatures of epithelial dysfunction and inflammation [38].

In an attempt to bridge this gap, providing further insights into bacterial taxa capable of converting lignans into EL/ED, here we investigated the lignan profile of a commercially prepared blend of oilseeds as a functional dietary supplement for women, by ultra-high-performance liquid chromatography–tandem mass spectrometry (UHPLC–MS/MS), and then used this as a substrate for inoculating stool samples from young and premenopausal women in in vitro anaerobic stirred batch culture systems, to simulate the physicochemical characteristics of the distal colon. These in vitro batch systems are technological platforms that can simulate the spatial, temporal and environmental features that microbes experience within the gut environment. Being host-free systems, these are the ideal systems in which to study microbial perturbations resulting from the addition of exogenous stimuli, as microbial changes can be measured in isolation of any concurrent effects on the host [39–45]. The aim of these experiments was to determine the production of oilseed mix-derived enterolignans and to explore differences in bacterial taxa that could be involved in lignan transformation. We investigated the dynamics of individual lignans (Seco, Lari, Pino and Mat) as well as enterolignans (ED and EL), gut microbial profiles and metabolic end products of microbial fermentation. The faecal-derived microbial communities were profiled at 0, 5 and 24 h by 16S rRNA gene-based next-generation sequencing, and the dietary lignan content was measured by UHPLC–MS/MS were profiled at 0.2, 5 and 24 h.

## Results

### Sample hydrolysis, extraction and quantification

The methodology for the extraction of lignans from food samples as well as of enterolignans from fermentation faecal samples was adapted from the work of Nørskov and Knudsen [46] as well as the work of Milder et al. [47] with minor modifications. The combination of solvent-based extraction and alkaline hydrolysis was used to release lignans from food or faecal matrix. On the other hand, enzymatic extraction with β-glucuronidase/sulfatase was performed in order to degrade the glycosidic bonds and to release the lignan aglycones. Solid Phase Extraction method was used to prepare samples for UHPLC–MS/MS analysis.

A UHPLC–MS/MS method was developed to obtain high resolution and signal accuracy. The deuterated standard Secoisolariciresinol-d^6^ (Seco-d6) was used as the reference standard. A multiple reaction monitoring (MRM) method was set up in negative ion mode for each analyte and quantification was established using the most intense MRM signal transition with 8–16 point calibration curves of pure analytical standards. The obtained MRM detection parameters are given in Table 1.

**Table 1:** Multiple reaction monitoring (MRM) detection parameters

Compound	MW	Parent m/z	fr m/z	CV	CE	Equation	R^2^	LOD	LOQ
Seco	362.42	361.4	122^a^	30	30	y = 5.5627x	0.9989	0.025571	0.085237
Seco	362.42	361.4	346		17				
Lari	360.4	359.4	329^a^	30	20	y = 20.963x	0.991	0.009145	0.030483
Lari	360.4	359.4	192		10				
Pino	358.39	357.4	151^a^	30	17	y = 9.5455x + 261.14	0.9914	0.011945	0.039818
Pino	358.39	357.4	136		30				
Mat	302.36	357.42	83^a^	30	23	y = 33.014x + 1235.7	0.9942	0.013987	0.046623
Mat	303.36	357.42	137		20				
ED	298.33	301	253^a^	30	20	y = 67.712x + 5969.6	0.9912	0.111075	0.370249
ED	298.33	301	271		20				
EL	298.33	297.3	107^a^	30	25	y = 105.71x + 17165	0.9984	0.019281	0.06427
EL	298.33	297.3	189		20				
Seco d^6^	368.45	367	168^a`^	30	25	y = 4.2692x − 60.534	0.9985	0.033394	0.111314
Seco d^6^	368.45	367	124		29				

*MW* Molecular weight, *fr* fragment; ^a^ quantification fragment; *CV* collision voltage, *CE* collision energy, *LOD* limit of detection; *LOQ* limit of quantification. Calibration curves showed good linearity, *LOD* was calculated as a signal to noise ratio (S/N) equal to 3; LOQ was calculated as a signal to noise ratio (S/N) equal to 10

### Determination of the lignan content in the oilseed mix

Obtained concentrations (mg/100 g of dry matter) of free and total (free plus bound) Seco, Lari, Pino and Mat in the oilseed mix are listed below in Table 2.

**Table 2:** Free and total (free plus bound) lignans in oilseed mix and baseline concentration in in vitro fermentation medium

	Mean/SD	Seco	Lari	Mat	Pino	Total
Oilseed mix lignans (mg/100 g)						
Free	Mean	31.1	44.9	3.8	10.7	90.5
	SD	9.2	19.1	0.4	2.3	30.9
Total	Mean	1467.6	188.9	132.2	172	1960.7
	SD	194.5	4.3	23.6	0.5	222.9
Lignan baseline concentration in the fermentation medium (ng/ml)						
Free	Mean	1480.95	2138.10	180.95	509.52	4309.52
	SD	438.1	909.5	19.0	109.5	1471.4
Total	Mean	69885.71	8995.24	6295.24	8190.48	93366.67
	SD	9261.9	204.8	1123.8	23.8	10614.3

As for the baseline in fermentation experiments, 0.5 g of oilseed mix was added to 105 ml of fermentation medium. Data are expressed as the mean ± SD (standard deviation)

The UHPLC–MS/MS analysis revealed that amongst free fractions, the quantities of Lari in the oilseed mix prevail (44.9 mg out of 90.5 mg per 100 g of mix), whereas amongst total lignans, the mean values for Seco are the highest (1467.6 mg out of 1960.7 mg per 100 g of mix).

### Analysis of enterolignans in fermented samples

**Fig. 2:**
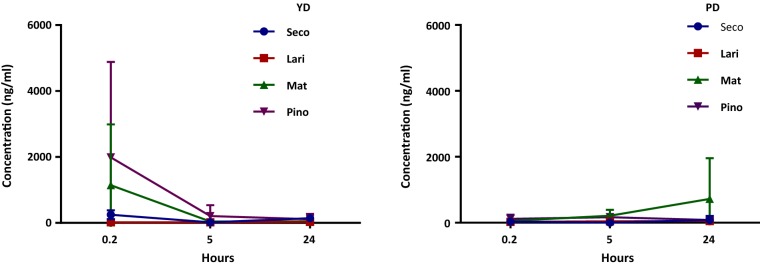
Dynamics of individual dietary lignans (Seco, Lari, Mat and Pino) over 24 h of fermentation in faecal samples from younger healthy donors (YD) and premenopausal donors (PD) with added oilseed mix. Data are expressed as the mean ± SD

**Fig. 3:**
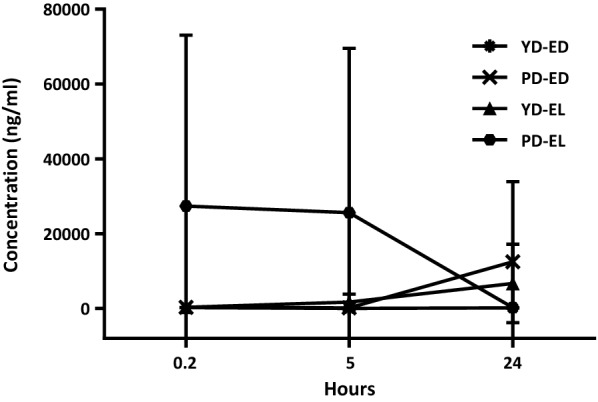
Concentration of enterodiol (ED) and enterolactone (EL) in faecal samples from younger healthy donors (YD) and premenopausal donors (PD) at 0.2 (T0.2), 5 (T5) and 24 h (T24) of fermentation with added oilseed mix

The results of batch culture fermentation experiments revealed differences in the concentration of individual lignans and enterolignans at different time points in the samples with added oilseed mix between young healthy (YD) and premenopausal (PD) donors (Table 3). Figures 2 and 3 report the values of free lignans and enterolignans respectively, assessed during the fermentation at 3 time points, 0.2 (T0.2), 5 (T5) and 24 h (T24).

**Table 3:** Concentration of lignans (Seco, Lari, Mat and Pino) and enterolignans (ED and EL) in the faecal samples from younger healthy donors and premenopausal donors at 0.2 (T0.2), 5 (T5) and 24 h (T24) of fermentation

	T0.2	T5	T24
Younger healthy donors			
Seco	246 ± 267.5	19.7 ± 21.6	142.7 ± 134.7
Lari	19.2 ± 19.1	18 ± 13.1	32.8 ± 30
Mat	1140.9 ± 1846.6	40.2 ± 20.6	45.8 ± 64.5
Pino	1987.8 ± 2899.2	207.3 ± 334.6	111.2 ± 163.3
ED	233 ± 227.5	38.7 ± 49.6	160.4 ± 166.7
EL	330.4 ± 397.4	1748.9 ± 2166.6	6739.2 ± 10554.4
Total (lignans)	3393.9 ± 2304.6	285.2 ± 389.9	332.5 ± 392.5
Total (enterolignans)	563.4 ± 445.8	2034.05 ± 2556.6	7071.7 ± 10946.9
Premenopausal donors			
Seco	30.52 ± 6.6	4.5 ± 1.82	81 ± 121.2
Lari	27.81 ± 8.9	43.3 ± 35.8	51.3 ± 38.1
Mat	51.3 ± 14.6	528.4 ± 580.9	743.1 ± 1088.9
Pino	124.1 ± 167.5	4.8 ± 6.6	12 ± 10.5
ED	247.13 ± 475.23	176.6 ± 183	12471.8 ± 21453.5
EL	20809.5 ± 55911.5	25619 ± 43962.5*	258.9 ± 226.7
Total (lignans)	233.66 ± 197.5	580.9 ± 625	887.3 ± 1258.7
Total (enterolignans)	21056.6 ± 56386.8	26199.9 ± 44587.5	1146.2 ± 1485.4

Data are expressed in ng/ml, as the mean ± SD (standard deviation). *p < 0.05, indicating a significant difference among samples collected after 0.2, 5 and 24 h of fermentation with the oilseed mix (Kruskal–Wallis test)

The average sum of the total individual lignans in the YD samples falls about 10 times during the first 5 h of fermentation, with a slight increase at 24 h. The concentration of Mat and Pino in this group decreased significantly, whereas Seco only slightly decreased and Lari increased. At the same time, the amounts of total enterolignans in the YD samples increased more than 10 times in 24 h, with EL expanding nearly 20 times but ED being slightly reduced.

In contrast, different dynamics were found in the fermentation samples from PDs: the total lignans increased almost 4 times in 24 h with only Pino falling about 10 times, while the levels of Seco, Mat and Lari expanded. Additionally, the amount of total enterolignans halved in 24 h, with ED levels expanding nearly 60 times, but EL dropping almost 100 times from 0.2 h to 24 h, suggesting the inability of faecal bacteria from PDs to convert ED to EL. It is also worth noting that all premenopausal participants entered the experiment with very different initial levels of enterolignans (particularly, ED), resulting in a high value of standard deviation.

Figure 2 shows the dynamics of Seco, Lari, Pino and Mat in the samples from YDs (a) and PDs (b) with added oilseed mix in 24-h fermentation experiments. While very different concentrations of lignans between YDs and PDs can be observed at T0.2, at T5 their levels are almost identical as well as at T24, except for Mat, whose level increased almost 500 times in the PD group.

Figure 3 illustrates the dynamics of ED and EL in YD and PD fermentation samples during the 24-h batch culture experiment. The ED levels are equally low in YD and PD samples at T0.2 and T5, while at T24, the value in the PD samples is almost 37 times higher than in the YD samples. As for EL, the average concentration is remarkably high in the PD samples at T0.2, falling slightly at T5 and experiencing a tremendous drop at T24. Conversely, the EL levels in the YD samples are insignificant at the beginning, slightly increase at T5 and are boosted at T24. Taken together, our results clearly demonstrate different dynamics of lignan transformation in different age groups of women: adding oilseed mix to YD samples leads to an overall drop in levels of dietary lignans and to the production of enterolignans. Contrary to this, in PDs, Mat levels increase after 24 h, with increased level of ED but decreased level of EL. These observations suggest that PDs may have a different, potentially altered intestinal microbiota configuration, with very poor conversion of dietary lignans and inability to efficiently convert ED or Mat to EL.

### Impact of the oilseed mix on faecal-derived microbial communities from younger healthy and premenopausal women

The faecal microbial communities from YDs and PDs were profiled over time during the fermentation experiments, to assess whether the different dynamics of conversion of the lignans of the oilseed mix were potentially attributable to different layouts and trajectories of the gut microbiota. In parallel, for each woman, two other batch cultures were set up, inoculated with the well-known prebiotic compound inulin (Raftilose P95) as a positive control or without addition of any compound (i.e. the negative control), respectively. The 16S rRNA gene-based next-generation sequencing of all fermentation samples yielded a total of 1,809,764 high-quality reads, with an average of 33,514 ± 7194 sequences per sample, binned into 2623 amplicon sequence variants (ASVs) at 99% similarity.

No significant differences were observed in alpha diversity across the entire dataset, regardless of the origin of the faecal sample (PD vs. YD), experimental condition (oilseed mix vs. inulin vs. negative control) and time point (T0 vs. T5 vs. T24) (p value > 0.05, Wilcoxon test). However, it should be noted that the biodiversity of both PD and YD faecal-derived microbial communities tended to decrease over time in all experimental conditions, except for the negative controls where it was generally kept (Additional file 1: Fig. S1). With specific regard to the oilseed mix, this decreasing trend was already appreciable after 5 h of fermentation in PD samples while only after 24 h for YD samples.

**Fig. 4:**
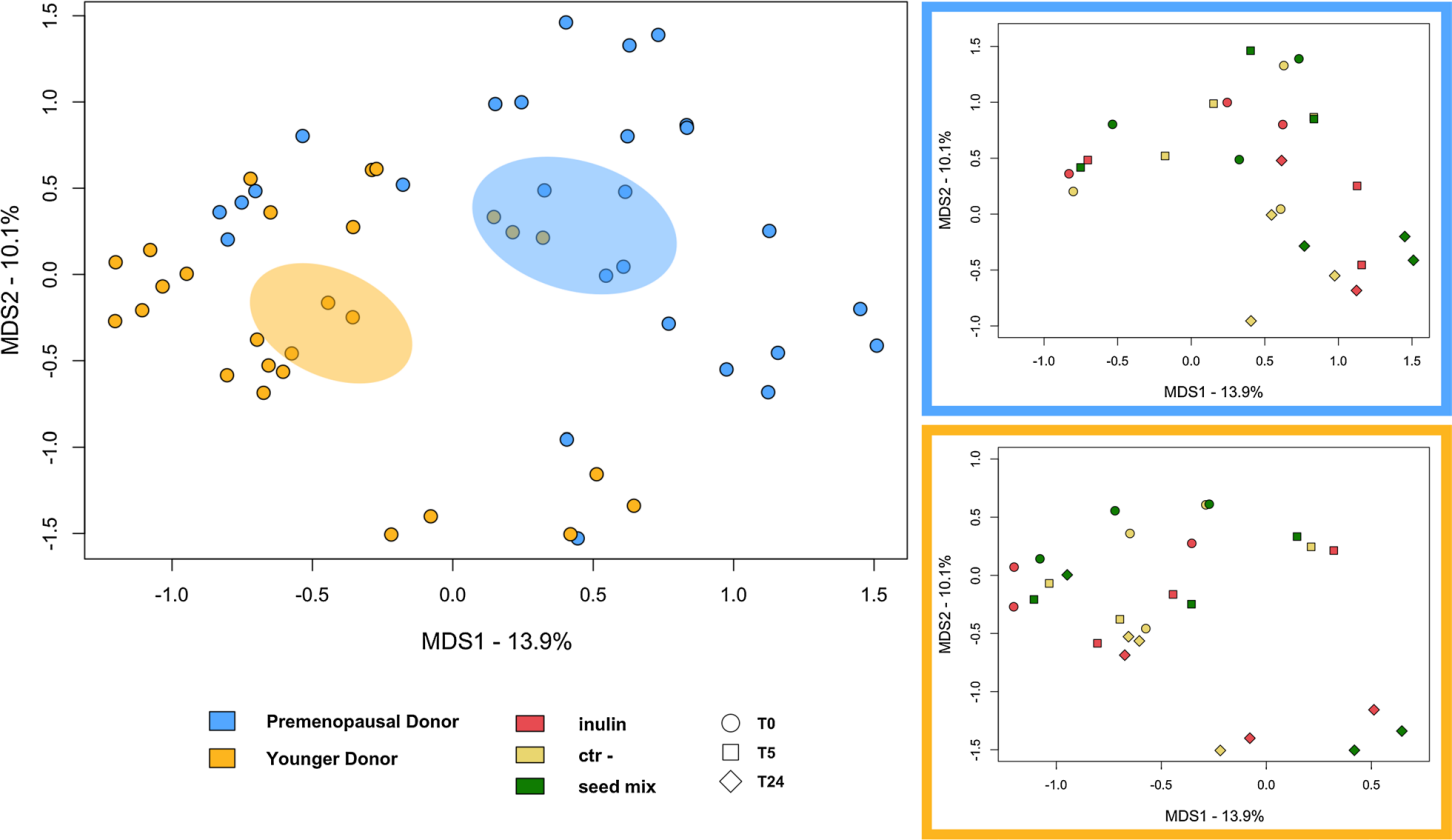
Faecal-derived microbial communities of premenopausal women separate from those of younger healthy women in 24-h fermentation experiments in the presence of oilseed mix, inulin or without additions. Left, Principal Coordinates Analysis (PCoA) based on unweighted UniFrac distances, showing all fermentation samples, coloured by group of women (premenopausal, light blue vs. younger healthy, orange). A significant separation between groups was found, regardless of experimental condition (oilseed mix, inulin and negative control - “Ctr-”) and time point (T0, T5 and T24) (p value < 1 × 10^−4^, permutation test with pseudo-*F* ratio). Right, PCoA plots showing the fermentation samples for premenopausal women (top panel) and younger healthy women (bottom panel). Within each group of women, the samples separate significantly by experimental condition (oilseed mix, green; inulin, red; Ctr-, yellow) and time point (T0, circle; T5, square; T24, diamond) (p value < 0.001)

The Principal Coordinates Analysis (PCoA) of inter-sample variation (i.e. beta diversity), based on unweighted (Fig. 4) and weighted (Additional file 2: Fig. S2) UniFrac distances, showed a significant separation between the faecal-derived microbial communities of PDs and YDs, regardless of the experimental condition and time point (p value < 1 × 10^−4^, permutation test with pseudo-*F* ratio). Within each group (PD and YD), the samples still segregated significantly according to both the experimental condition and the time point (p value < 0.001), suggesting a differential impact of supplements over time, likely related to the baseline microbial community. In particular, the PD faecal microbial ecosystem underwent an early modification of its overall structure, as evidenced by the shift at T5, and continued to evolve up to 24 h under all experimental conditions. The same was basically true for YD samples based on unweighted UniFrac distances, while a significant shift in the weighted UniFrac-based PCoA was observed only at T24, suggesting a dominant configuration likely to be more resilient to external perturbations compared to the PD one. Please, see Additional file 3: Table S1 for the results of adonis and ANOSIM statistics applied to weighted and unweighted UniFrac distance-based ordination.

At T0, a number of significant taxonomic differences were indeed observed between PDs and YDs. In particular, compared to YDs, the baseline faecal-derived microbial ecosystem of PDs showed increased relative abundance of some Bacteroidetes members, especially *Bacteroides* and *Rikenellaceae*, as well as *Ruminococcaceae* genera (p value ≤ 0.05, Wilcoxon test). On the other hand, *Coriobacteriaceae*, especially *Collinsella*, and *Streptococcus* were much more represented in the baseline microbiota of YDs vs. PDs (p value ≤ 0.05) (Additional file 4: Fig. S3).

**Fig. 5:**
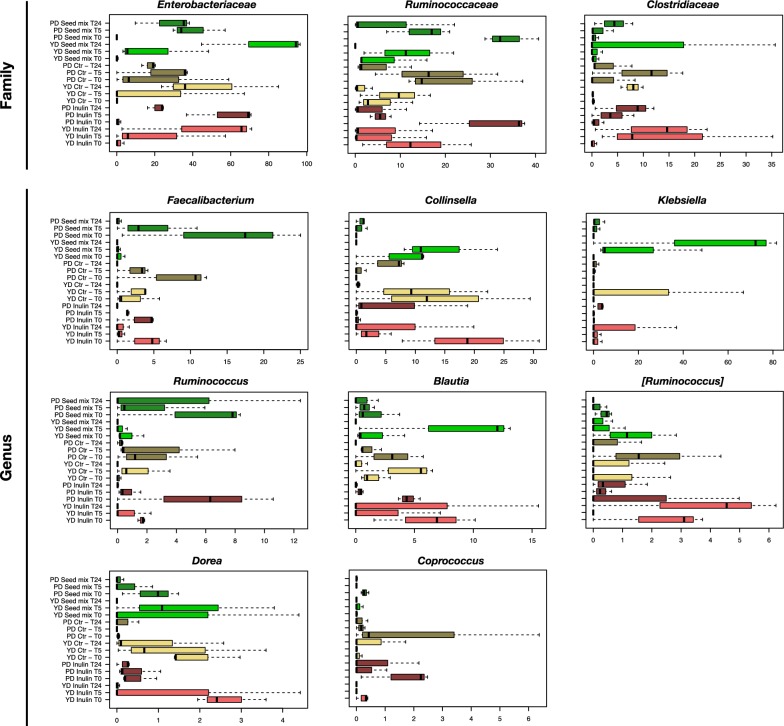
Families and genera of the faecal-derived microbial communities of premenopausal (PD) and younger healthy women (YD), differing over time in 24-h fermentation experiments in the presence of oilseed mix, inulin or without additions (Ctr -). Boxplots showing the relative abundance distribution of bacterial taxa in the different study groups at T0, T5 and T24 (YD inulin, light red; YD Ctr -, light yellow; YD oilseed mix, light green; PD inulin, dark red; PD Ctr -, dark yellow; PD oilseed mix, dark green). Only significantly different taxa or trends are shown (p value ≤ 0.2, Wilcoxon test)

With specific regard to the impact of oilseed mix on the faecal-derived microbial communities of PDs vs. YDs, we observed both common and unique microbial signatures of response (Fig. 5). Among the features shared between PDs and YDs, it is worth noting that 24 h of fermentation with the oilseed mix resulted in decreased proportions of *Ruminococcaceae* and various members of *Lachnospiraceae*, and increased amounts of *Enterobacteriaceae* (p value ≤ 0.1). Although in the absence of statistical significance, these variations were also observed in the control vessels (i.e. with the addition of inulin or without any supplement), suggesting that they could be related to the experiment per se. Nonetheless, the *Enterobacteriaceae* increase in the 24-h faecal-derived microbial communities of YDs in the presence of the oilseed mix was far greater than in PDs (p value = 0.012) (24-h relative abundance: YDs with the oilseed mix, 78.5%; PDs with the oilseed mix, 27.7%; YDs with inulin, 46.4%; YDs without addition, 48.2%; PDs with inulin, 21.5%; PDs without addition, 17.5%). Going down in the taxonomic classification, we found that this increase was largely attributable to *Klebsiella*, whose 24-h relative abundance was 51.3 vs. 1.7% in the YD and PD group, respectively. The mean values in the YD and PD controls were 6.2% (± 8.7%, SD) and 1.7% (± 1.3%), respectively. Unfortunately, the other enterobacteria were unclassified at the genus level. At the species level, *Klebsiella* ASVs were found to be variously assigned to *K. pneumoniae* and *K. aerogenes*, with the latter being overall more prevalent (i.e. present in 9/27 fermentation samples of YDs, of which 5 in the presence of oilseed mix, while only in 5/27 for PDs, of which 3 in the presence of oilseed mix). Furthermore, it is interesting to point out that the family *Clostridiaceae* increased in PD-derived microbial communities in the presence of oilseed mix but not in the YD-related ones, as well as the genus *Collinsella*, whose trend was exactly opposite (i.e. decreased) in YD-related samples (p value ≤ 0.2).

## Discussion

In this work, we determined the concentration of four main dietary lignans in an oilseed mix commercially prepared from the following ingredients: pumpkin, sunflower seeds, buckwheat and millet flakes, milled flaxseed, hemp and chia. Compared to flaxseed, the oilseed mix had 4 times less free lignans with Seco being 8.2 times less and Mat almost 6 times less than in flaxseed. Comparable volumes of Lari were found in both flaxseed and oilseed mix: 44.2 mg/100 g and 44.9 mg/100 g, respectively. Seco and Mat are the main substrate for enterolignans, with Seco being converted to EL via ED, and Mat being directly converted to EL. Differences in lignan concentration have been previously discussed [5, 48] and are thought to be related to variance in crop, climate, storage conditions and other factors. Therefore, it was expected to obtain concentrations that would diverge from previously reported data.

We then used the oilseed mix in batch culture fermentation experiments to evaluate the dynamics of enterolignan production by faecal-derived microbial communities from younger healthy (YD) vs. premenopausal (PD) female donors [49]. In vitro fermentation systems have been used for many years, however most models have yet to fully characterize the reproducibility (technical replicates), repeatability (biological replicates), and stability (time to and maintenance of enviromental conditions) of cultured communities [39–45].

Adding 0.5 g of oilseed mix to the stool samples of YDs resulted in a tenfold increase in EL concentration and a decrease in ED in 24 h, as compared to the negative control. Contrary to this, the same amount of supplement in the presence of PD samples resulted in a significant ED boost, whose 24-h concentration increased 110 times compared to the negative control and 80 times compared to the YD group under the same experimental conditions. However, despite such a high volume of ED, EL levels continued to decline, probably indicating the failure of bacterial communities from PDs to efficiently convert ED to EL. Consistent with this, i.e. with the inability of PD-related microbiotas to produce EL, high levels of Mat were found at the end of fermentation [50, 51].

The biotransformation of dietary lignans into EL and ED is a complex process believed to involve microbial consortia capable of performing four major catalytic reactions—*O*-deglycosylation, O-demethylation, dihydroxylation, and dehydrogenation—with each reaction being catalyzed by a specialized group of bacteria that often constitute a minor component of the overall microbiome [34–36]. Although evidence has been provided of the association between specific enterolignans and certain human gut bacteria, such as that between high urinary EL excretion and increased proportions of *Ruminococcus* spp. while decreased amounts of *Bilophila* [36, 52], the complexity and diversity of the gut microbiota are considered essential to maximise lignan conversion and thus influence human exposure to enterolignans. In this regard, here we demonstrated that YDs and PDs have a different intestinal microbiota profile and that these profiles undergo different temporal dynamics in the presence of oilseed mix, potentially contributing to the different production pattern of EL/ED, as observed in our in vitro batch culture fermentations. In particular, the early changes observed in the dominant structure of the faecal-derived microbial ecosystem of PDs seem to suggest a less resilient configuration and therefore more susceptible to disturbances, compared to YDs. From a taxonomic point of view, PDs were found to be significantly depleted in *Collinsella*, while being enriched in a number of Bacteroidetes members, including *Bacteroides* and *Rikenellaceae*, that typically thrive on high-fat diets and are enriched in obese gut microbiomes [53]. As regards the impact of the oilseed mix, both YD and PD faecal-derived microbial communities showed an enrichment over time in *Enterobacteriaceae*, specifically *Klebsiella*, but with a 24-h abundance much greater in the former. As previously observed [54, 55], several *Klebsiella* spp.—closely related to *K. pneumoniae*—are capable of biotransforming SDG into Seco, with the latter representing a substrate for EL production through microbial metabolism. The differential representation of *Klebsiella* could therefore help explain the greater levels of EL in YD- vs. PD-related samples. On the other hand, it is worth noting the differential trend of *Collinsella*, whose relative abundance increased in PD-related microbial communities while it decreased in YD-related ones in the presence of oilseed mix, which suggests a possible role of this taxon in EL/ED conversion. Interestingly, *Collinsella* has recently been described as a bacterial genus involved in the biotransformation of dietary phytoestrogens in equol, possibly contributing to the desirable health benefits of isoflavones in postmenopausal women [56, 57]. Finally, the PD faecal-derived microbial ecosystem appeared to be enriched in the *Clostridiaceae* family, whose members are already known to be variously involved in lignan metabolism [58, 59].

## Conclusion

Despite the small sample size and obvious limitations of an in vitro study, our findings support that the faecal microbiota of healthy younger women is more efficient in converting dietary lignans into enterolignans, especially EL, than the microbiota of older premenopausal women. Furthermore, our study points to *Klebsiella* and *Collinsella* spp. as previously overlooked actors of the human gut microbiota with a strong potential to be directly involved in EL/ED production. To this end, in vitro gut microbiota models have proved to be useful tools to study the effects of food components, diet, and pharmaceutical molecules on gut microbiota composition. The advantages and limitations of in vivo and in vitro models as well as the developments to improve the modeling of host-microbe interactions have been extensively reviewed. Future in vivo studies in larger cohorts are needed to confirm our findings and understand how we can modulate the microbiota composition, favouring microbial species capable of producing EL more effectively. If a woman’s microbiota effectively converts dietary lignans to EL, consistent consumption of lignan-rich foods will potentially preserve a woman’s health in the long term, preventing or at least delaying the onset of degenerative conditions typically associated with menopause.

## Methods

### Oil seed samples

Eurocan Ltd (UK) provided five different organic raw oil seeds (flaxseed, chia, hulled sunflower, hulled pumpkin, hulled hemp), organic buckwheat and millet flakes, as well as a ready-made oilseed mix “MightyMix”. Oilseed mix was milled using Hinari Genie MB280 electric grinder. Duplicates 25 mg of the mix were stored in separate 2-ml Eppendorf tubes at − 20 °C. The present oilseed mix nutritional profile was characterised by Campden BRI (Chipping Campden) Ltd, accredited to ISO17025:2005 by UKAS by official reference methods (fat: Weibull Stoldt; sugars: HPLC; total dietary fibre: AOAC; protein: Kehjdahl) as reported in Additional file 5: Table S2.

### Stool sample collection and preparation

Faecal samples were donated by three healthy younger (aged 25–30 years) and three older premenopausal female donors (aged 40–55 years). All donors confirmed to be healthy of metabolic and gastrointestinal conditions, were not taking prebiotic or probiotic supplements and did not receive antibiotic treatments in the 6 months preceding the study. Information on the donors’ health status, lifestyle habits, clinical anamnesis, and medicine use was collected with a pre-informative questionnaire. All faecal samples were collected on site, kept at − 20 °C and used within a maximum of 15 min after collection. The samples were diluted 1/10 w/v in anaerobic PBS (0.1 mol/l phosphate buffer solution, pH 7.4) and homogenized (Stomacher 400, Seward, West Sussex, UK) for 2 min at 460 paddle-beats.

### In vitro batch culture fermentation experiments

The batch culture fermentation method was carried out as previously described by Gibson et al. [39]. Each vessel was inoculated with 5 ml of fresh faecal slurry (1/10 w/v) for both healthy and premenopausal subjects. A known prebiotic compound inulin (Raftilose P95, 95% oligosaccharide, β(2-1)-fructan, of which 60% w/w glucose-fructose, 40% w/w fructose, degree of polymerization, 3–10) serving as a positive control was added to a separate batch-culture vessel. A further vessel was prepared under the same conditions but without the addition of any compound (negative control, “ctr-”) whereas another vessel was used to add the oilseed mix. Batch culture fermenters were ran under anaerobic conditions for a period of 24 h during which samples (5 ml) were collected at time 0, 0.2, 5 and 24 h. Specifically, T0 samples for microbiota analysis were taken from the respective vessels under operating conditions and not from the women’s feces. Samples were stored at − 80 °C until needed for 16S rRNA gene-based next-generation sequencing analysis and ultra‐high-performance liquid chromatography (UHPLC)–tandem mass spectrometry (MS/MS) quantification.

### Lignan extraction methods

The methodology for the extraction of lignans from single food samples and enterolignans from faecal samples was adapted from the work of Nørskov and Knudsen [46] as well as Milder et al. [47] and optimised in regards to the weight and the character of the samples. To each oilseed sample (25 mg), 1 ml of *n*-hexane, was added. The samples were vortexed and left at room temperature with gentle agitation for 20 min. Samples were centrifuged at 13,200 rpm, at 4 °C for 10 min. The supernatant was discarded, and the pellets were kept for the next steps.

### Extraction of free lignans from oilseed samples and fermentation samples

#### Oilseed free lignan samples

Pellets were extracted with 0.5 ml of 100% methanol, vortexed and left at room temperature with gentle agitation for 10 min. After centrifugation at 13,200 rpm, at 4 °C for 10 min, the supernatant was collected into clean 2-ml Eppendorf tubes and left at nitrogen stream to evaporate for dryness.

#### Fermentation enterolignan samples

One milliliter of fermentation sample was extracted with 0.5 ml of 100% methanol, sonicated for 10 min, and then kept with gentle agitation for 10 min. Next, samples were centrifuged at 13,200 rpm, for 10 min at 4 °C, and the supernatant was collected into clean 2-ml tubes and evaporated for dryness.

Afterwards, dried fermentation and oilseed samples were incubated for 16 h at 37 °C with added ß-glucuronidase/sulfatase from Helix pomatia (freshly dissolved in 0.05 M sodium acetate buffer with concentration of 2 mg/ml), cooled and added with 0.5 ml of acidified water (0.4% of formic acid) to stop the hydrolysis, vortexed and then centrifuged at 13,200 rpm for 10 min at 4 °C. Samples were ready for solid phase extraction (SPE).

### Extraction of total lignans from oilseed samples

Following defatting, pellets were extracted with 0.5 ml of 0.3 M NaOH in 70% methanol, vortexed, and then incubated for 1 h with gentle agitation at 60 °C. After cooling down, the pH was adjusted by adding 20 µl of glacial acetic acid (pH = 5). Next, samples were centrifuged for 10 min at 13,200 rpm at 4 °C; the supernatant was collected into 2-ml clean Eppendorf tubes and evaporated for dryness under a nitrogen stream. Enzymatic hydrolysis was performed by adding 0.6 ml of ß-glucuronidase/sulfatase from Helix pomatia (same as above) to each dried sample and setting samples for overnight incubation at 37 °C coupled with gentle agitation. Afterwards, samples were cooled down, added with 0.5 ml of acidified water (0.4% of formic acid), vortexed and then centrifuged at 13,200 rpm, at 4 °C for 10 min. Supernatants were collected into clean Eppendorf tubes ready SPE.

### Solid Phase Extraction (SPE) of lignans and enterolignans

SPE was performed using 1-ml cartridges Strata^®^ C-18 (55 µm, 70Å) from Phenomenex UK and SPE Vacuum Manifold. Waste was collected into 15-ml Falcon tubes, and final samples were collected into clean 2-ml Eppendorf tubes. After assembling manifold, cartridges were prepared as follows: with locked taps, 0.5 ml of acetonitrile was added to each cartridge and left for 10 min, then drained out. Next, with locked taps, 0.5 ml of LC–MS water was added, then drained after 10 min. After this, samples were added for slow elution through C18 material, followed by 0.5 ml of methanol, added twice to each cartridge and let to elute slowly until dry, afterwards, the vacuum was applied to dry the sorbent. Each cartridge was eluted with 0.4 ml of acetonitrile, and after draining, the vacuum was applied to facilitate full elution. Samples were then evaporated under a nitrogen stream and stored at − 20 °C. Aliquots of 0.5 ml of LC–MS water containing the internal standard (seco-d^6^) at the final concentration of 20 ng/ml and fermentation samples were added with 100 µl of LC–MS water. Samples were vortexed and 200 µl was dispersed into well plates ready for LC–MS analysis.

### Microbial DNA extraction

Total microbial DNA was extracted from around 250 mg of in vitro fermentation samples using the QIAamp DNA Stool Mini Kit (QIAGEN, Hilden, Germany) according to the manufacturer’s instructions. DNA concentration and quality were evaluated using the NanoDrop ND-1000 spectrophotometer (NanoDrop Technologies, Wilmington, DE).

### 16S rRNA gene-based next-generation sequencing, bioinformatics and statistics

The V3–V4 hypervariable region of the 16S rRNA gene was PCR-amplified using the primer set 341F/805R, as previously reported [60]. PCR products of about 460 bp were purified using a magnetic bead-based system (Agencourt AMPure XP; Beckman Coulter, Brea, CA) and indexed by limited-cycle PCR using Nextera technology (Illumina, San Diego, CA). Indexed libraries, further cleaned up as described above, were pooled at equimolar concentration, denatured and diluted to 6 pmol/l. Sequencing was performed on an Illumina MiSeq platform using the 2 × 250 bp paired-end protocol, according to the manufacturer’s instructions. Sequencing reads were deposited in the National Center for Biotechnology Information Sequence Read Archive (NCBI SRA; BioProject ID PRJNA592433).

The obtained paired-end reads were processed using a pipeline combining PANDAseq [61] and QIIME 2 [62]. High-quality reads were filtered and clustered into amplicon sequence variants (ASVs) at 99% similarity through an open-reference strategy performed with DADA2 [63]. Taxonomy was assigned using the vsearch classifier [64] against Greengenes database as a reference (release May 2013). Alpha diversity was measured using the number of observed ASVs and the Faith’s Phylogenetic Diversity (PD whole tree). Beta diversity was computed based on weighted and unweighted UniFrac distances and visualized on a Principal Coordinates Analysis (PCoA) plot. For the identification of *Klebsiella* species, the ASVs assigned to the genus *Klebsiella* were subjected to BLAST analysis [65]. Statistics was performed using R Studio 1.0.44 on R software version 3.3.2 [66] implemented with the packages stats and vegan [67]. The significance of data separation in the PCoA plot was tested by a permutation test with ANOSIM and pseudo-*F* ratio statistics using the function adonis in vegan. Bar plots were built using the R packages made4 [68] and vegan. Non-parametric tests (Kruskal–Wallis test or Wilcoxon test, paired or unpaired as needed) were achieved using the stats package. A p value ≤ 0.05 was considered statistically significant; a p value between 0.05 and 0.2 was considered a tendency.

### Ultra‐high-performance liquid chromatography (UHPLC)–MS/MS

Acquity H class UPL chromatography equipment was used and separations were performed on an Aquity UPLC^®^ HSS PFP 1.8 µm 2.1 × 100 mm C18 (Waters, UK) class column with a column protection of Acquity UPLC^®^ HSS T3 1.8 µm Van Guard™ pre-column 3/Pk 2.1 × 5 mm column (Waters, UK) at a flow rate of 0.65 ml/min at 30 °C. The mobile phases A and B consisted of 100% LC–MS water and 100% acetonitrile, respectively. The gradient started at 95% phase A and 5% phase B, was held constant for 6 min, then phase B increased to 75% during 0.9 min, with the subsequent increase to 95% for 0.1 min, followed by 99% increase of phase A during the last 2 min. The total run for each sample was 10 min. Sample injection volume was 2 µl. The negative mode was used for ionisation. Detection was performed using Xevo TQ-micro (Waters, UK) quadrupole mass spectrometer, which facilitates the detection of low concentrated analytes. Parent and daughter ions (m/z) are described for each compound in Table 1 together with cone voltage and collision energy. The data were collected and analysed using MassLynx software.

## Supplementary information

**Additional file 1: Fig. S1.** The biodiversity of the faecal-derived microbial communities from premenopausal and younger healthy women tends to decrease over time in 24-h fermentation experiments in the presence of oilseed mix and inulin. Boxplots showing the distribution of alpha diversity values, according to the number of observed amplicon sequence variants (ASVs, upper panel) and Faith’s Phylogenetic Diversity index (PD whole tree, lower panel), for the faecal microbial communities from premenopausal (PD) and younger healthy (YD) women at 0 (T0), 5 (T5) and 24 h (T24) of fermentation in the presence of oilseed mix (shades of green), inulin (as a positive control; shades of red) or without additions (negative control, “Ctr -”; shades of yellow).

**Additional file 2: Fig. S2.** Weighted UniFrac-based Principal Coordinates Analysis (PCoA) of the faecal-derived microbial communities of premenopausal and younger healthy women over 24 h of fermentation in the presence of oilseed mix, inulin or without additions. Left, PCoA plot showing all fermentation samples, coloured by group of women (premenopausal, light blue vs. younger healthy, orange). A significant separation between groups was found, regardless of experimental condition (oilseed mix, inulin and negative control—“Ctr-”) and time point (T0, T5 and T24) (p value < 1 × 10^−4^, permutation test with pseudo-*F* ratio). Right, PCoA plots showing the fermentation samples for premenopausal women (top panel) and younger healthy women (bottom panel). Within each group of women, the samples separate significantly by experimental condition (oilseed mix, green; inulin, red; Ctr-, yellow) and time point (T0, circle; T5, square; T24, diamond) (p value < 0.001).

**Additional file 3: Table S1.** Results of adonis and ANOSIM statistics applied to weighted and unweighted UniFrac distance-based ordination (related to Fig. 4 and Additional file 2: Fig. S2). PD: premenopausal donor; YD: younger donor.

**Additional file 4: Fig. S3.** Genus-level relative abundance profiles of the faecal-derived microbial communities of premenopausal (PD) and younger healthy women (YD) at 0, 5 and 24 h of fermentation in the presence of oilseed mix, inulin or without additions. *, unclassified amplicon sequence variants reported at higher taxonomic level; **, other. For each group of women (YD and PD), the profiles are shown in the following order: samples in the presence of inulin at T0, T5 and T24 (red), samples in the negative control (Ctr -) at T0, T5 and T24 (yellow), and samples in the presence of oilseed mix at T0, T5 and T24 (green). The black arrow below the histograms indicates the temporal succession for each triplet of samples, i.e. T0, T5, T24.

**Additional file 5: Table S2.** Composition and nutritional profile of the seed mix (MightyMix) used in the present study.

## Data Availability

The datasets used and/or analyzed during the current study are available from the corresponding author on reasonable request. Sequencing reads were deposited in the National Center for Biotechnology Information Sequence Read Archive (NCBI SRA; BioProject ID PRJNA592433).

## Abbreviations

SDG: Secoisolariciresinol diglucoside
Seco: Secoisolariciresinol
Lari: Lariciresinol
Pino: Pinoresinol
Mat: Matairesinol
ED: Enterodiol
EL: Enterolactone
ER: Oestrogen receptor
SPE: Solid phase extraction
LC–MS: Liquid chromatography–mass spectrometry
MRM: Multiple reaction monitoring
UHPLC: Ultra-high-performance liquid chromatography
YD: Younger donor
PD: Premenopausal donor
