# Supplementary figures and images for: Impact of lignans in oilseed mix on gut microbiome composition and enterolignan production in younger healthy and premenopausal women: an in vitro pilot study
Source: Microb Cell Fact. 2020 Apr 3;19:82. doi: 10.1186/s12934-020-01341-0 (PMC7119089; doi:10.1186/s12934-020-01341-0)

**Observed ASVs**

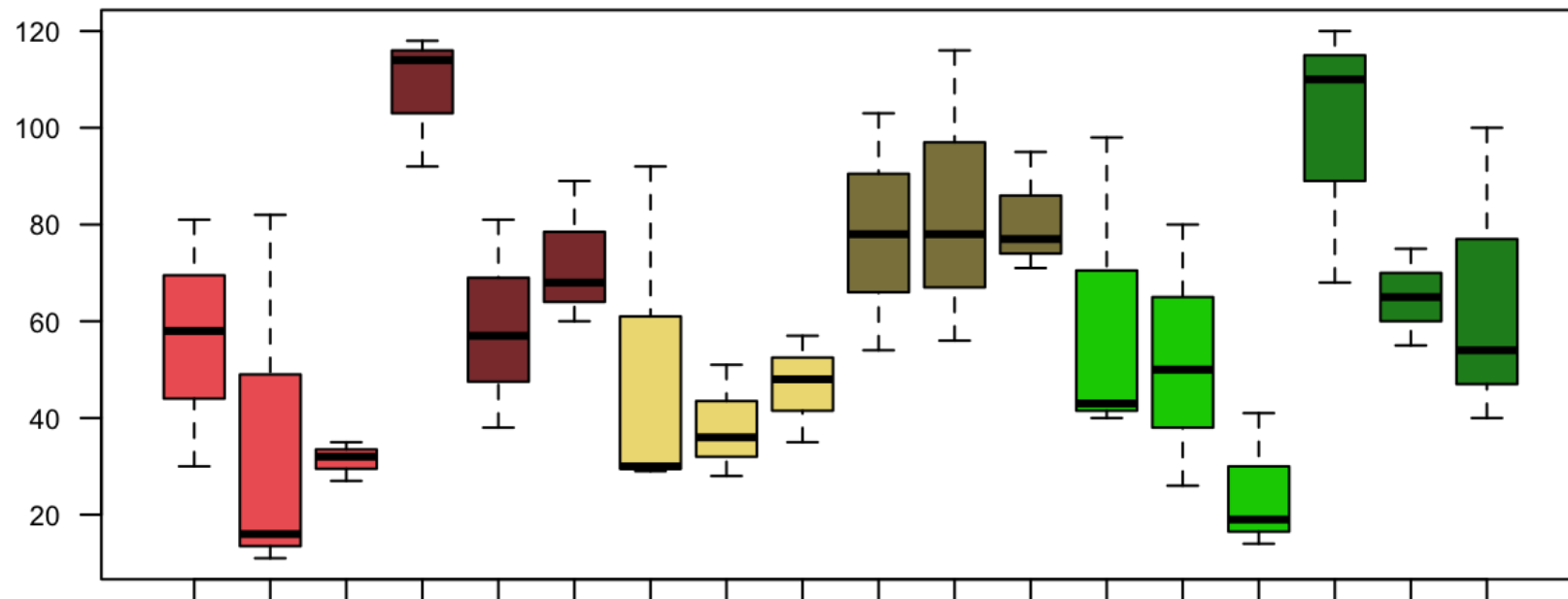

**PD whole tree**

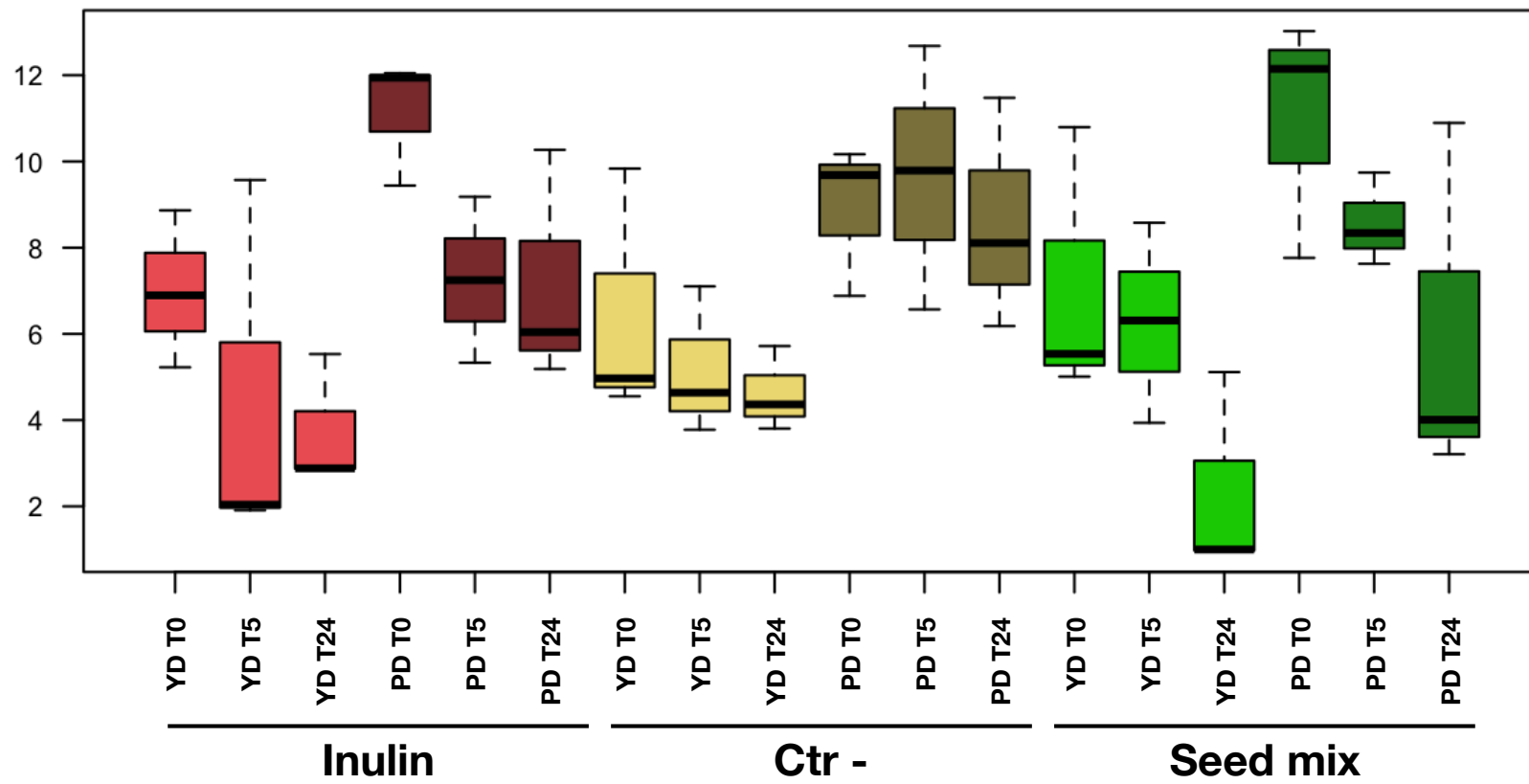

Supplement: Supplementary file 1 — Additional file 1: Fig. S1. The biodiversity of the faecal-derived microbial communities from premenopausal and younger healthy women tends to decrease over time in 24-h fermentation experiments in the presence of oilseed mix and inulin. Boxplots showing the distribution of alpha diversity values, according to the number of observed amplicon sequence variants (ASVs, upper panel) and Faith’s Phylogenetic Diversity index (PD whole tree, lower panel), for the faecal microbial communities from premenopausal (PD) and younger healthy (YD) women at 0 (T0), 5 (T5) and 24 h (T24) of fermentation in the presence of oilseed mix (shades of green), inulin (as a positive control; shades of red) or without additions (negative control, “Ctr -”; shades of yellow). [file 12934_2020_1341_MOESM1_ESM.pdf]

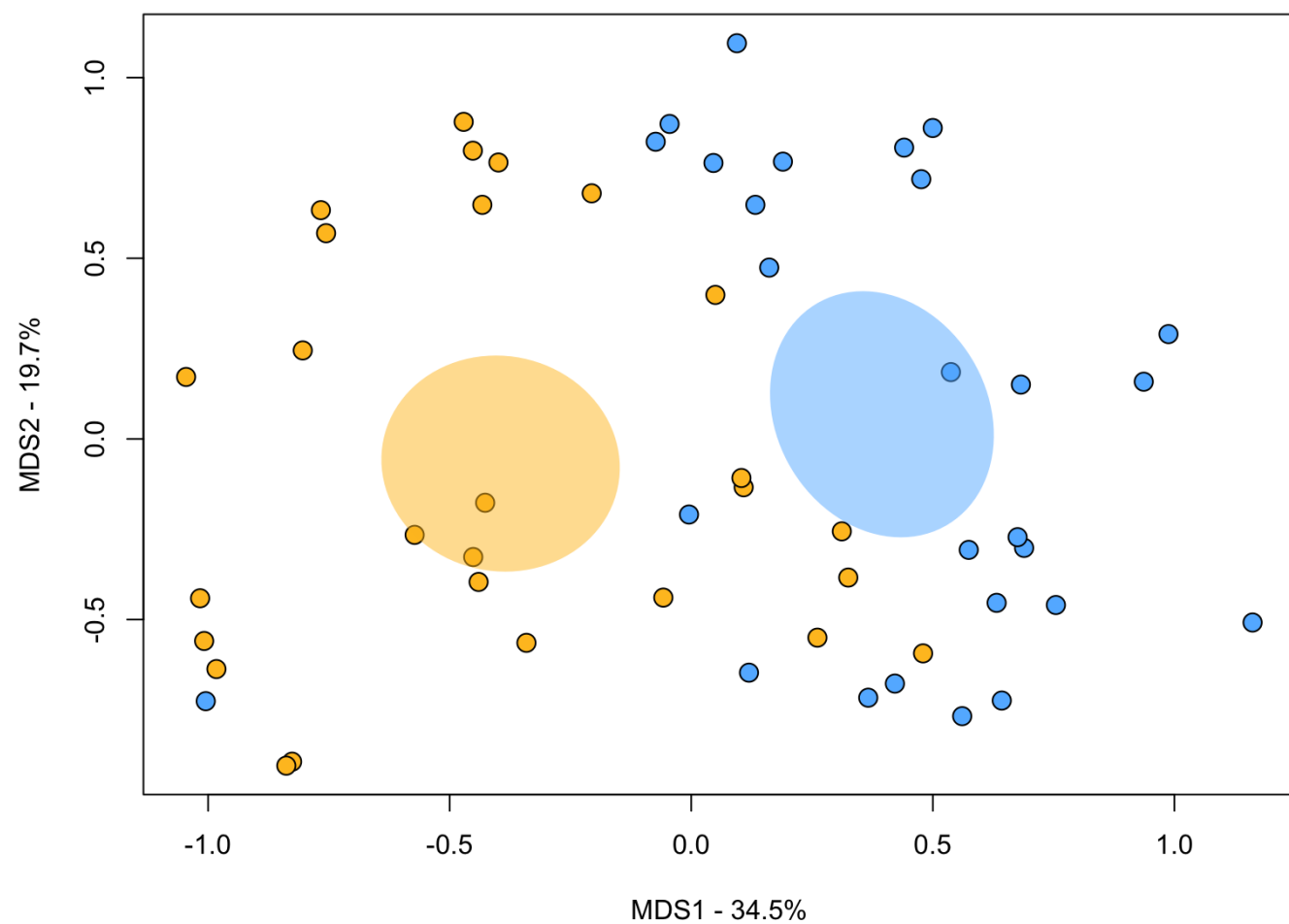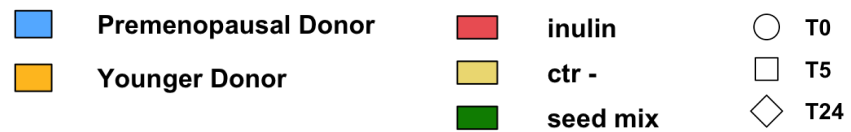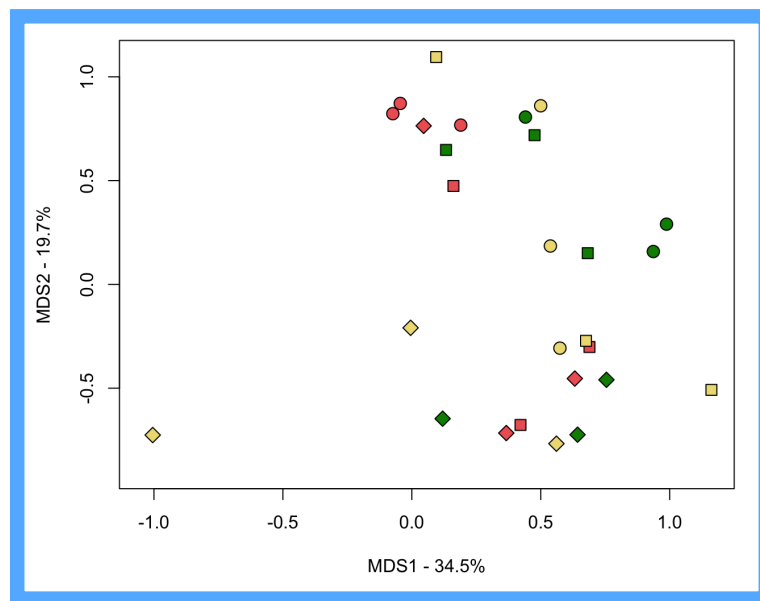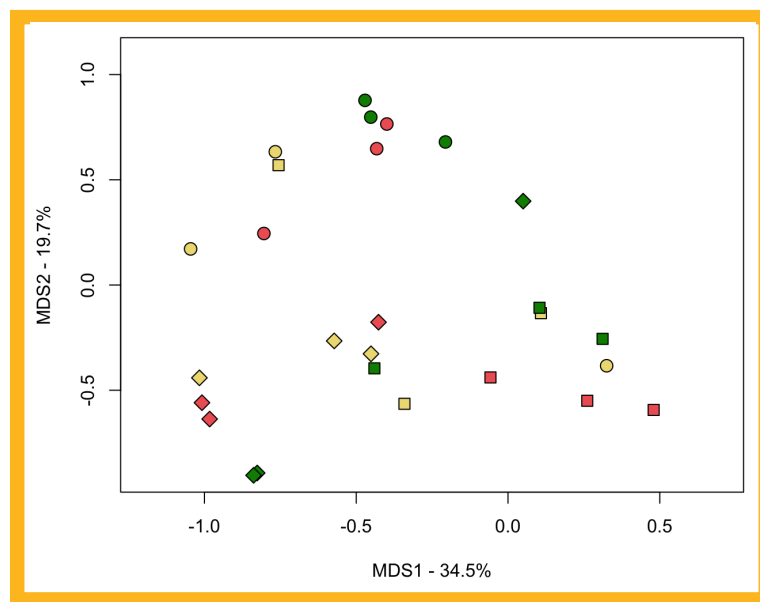

Supplement: Supplementary file 2 — Additional file 2: Fig. S2. Weighted UniFrac-based Principal Coordinates Analysis (PCoA) of the faecal-derived microbial communities of premenopausal and younger healthy women over 24 h of fermentation in the presence of oilseed mix, inulin or without additions. Left, PCoA plot showing all fermentation samples, coloured by group of women (premenopausal, light blue vs. younger healthy, orange). A significant separation between groups was found, regardless of experimental condition (oilseed mix, inulin and negative control—“Ctr-”) and time point (T0, T5 and T24) (p value < 1 × 10^−4^, permutation test with pseudo-F ratio). Right, PCoA plots showing the fermentation samples for premenopausal women (top panel) and younger healthy women (bottom panel). Within each group of women, the samples separate significantly by experimental condition (oilseed mix, green; inulin, red; Ctr-, yellow) and time point (T0, circle; T5, square; T24, diamond) (p value < 0.001). [file 12934_2020_1341_MOESM2_ESM.pdf]

# Relative abundance

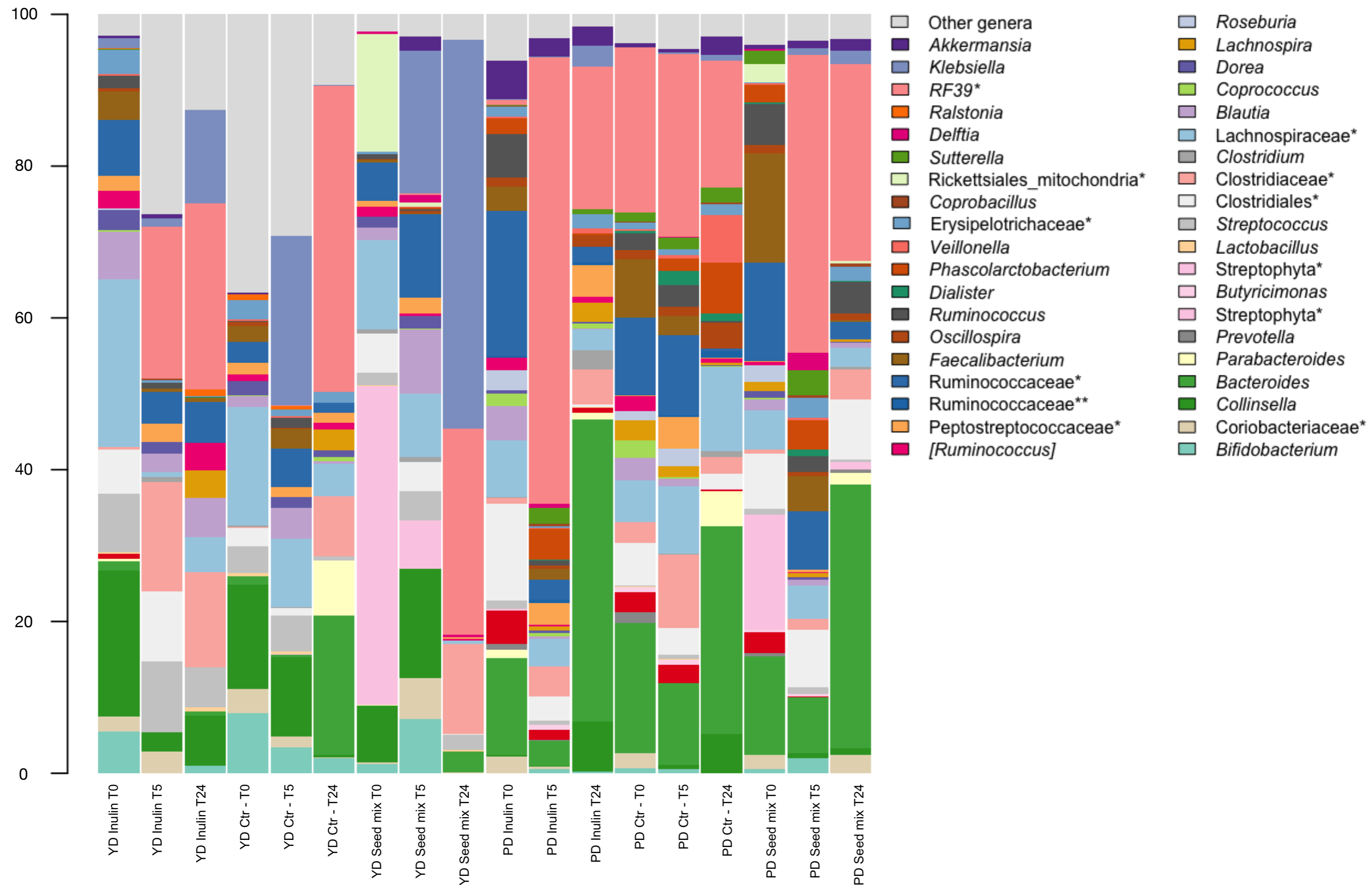

Supplement: Supplementary file 4 — Additional file 4: Fig. S3. Genus-level relative abundance profiles of the faecal-derived microbial communities of premenopausal (PD) and younger healthy women (YD) at 0, 5 and 24 h of fermentation in the presence of oilseed mix, inulin or without additions. *, unclassified amplicon sequence variants reported at higher taxonomic level; **, other. For each group of women (YD and PD), the profiles are shown in the following order: samples in the presence of inulin at T0, T5 and T24 (red), samples in the negative control (Ctr -) at T0, T5 and T24 (yellow), and samples in the presence of oilseed mix at T0, T5 and T24 (green). The black arrow below the histograms indicates the temporal succession for each triplet of samples, i.e. T0, T5, T24. [file 12934_2020_1341_MOESM4_ESM.pdf]
