# Supplementary material for: Impact of lignans in oilseed mix on gut microbiome composition and enterolignan production in younger healthy and premenopausal women: an in vitro pilot study
Source: Microb Cell Fact. 2020 Apr 3;19:82. doi: 10.1186/s12934-020-01341-0 (PMC7119089; doi:10.1186/s12934-020-01341-0)
Supplement: Supplementary file 5 — Additional file 5: Table S2. Composition and nutritional profile of the seed mix (MightyMix) used in the present study. [file 12934_2020_1341_MOESM5_ESM.docx]

| **Composition** | **Results** |  |
| --- | --- | --- |
| Energy (kcal) | 494 kcal/100 g |  |
| Protein | 21.3 g/100 g |  |
| Carbohydrate (Total) | 36.8 g/100 g |  |
| Sugars (Total)* | 0.9 g/100 g |  |
| Dietary Fibre (Total) | 8.7 g/100 g |  |
| Fat | 31.1 g/100 g |  |
| Fatty acids saturated | 4.2 g/100 g |  |
| Fatty acids monounsaturated | 8.2 g/100 g |  |
| Fatty acids polyunsaturated | 17.2 g/100 g |  |
| Fatty acids omega 3 | 4.6 g/100 g |  |
| Fatty acids omega 6 | 12.6 g/100 g |  |
| Fatty acids omega 9 | 8.1 g/100 g |  |
| Fatty acids ALA | 4.6 g/100 g |  |
| Fatty acids EPA | <0.1 g/100 g |  |
| Fatty acids DHA | <0.1 g/100 g |  |
| Moisture | 7.6 g/100 g |  |
| Ash | 3.29 g/100 g |  |

*Total sugars are the sum of glucose, sucrose and fructose expressed as monosaccharides
